# Supplementary material for: Coping mechanisms for students with mental disorders: An exploratory qualitative study at Busitema University’s Mbale and Busia Campuses
Source: PLOS Glob Public Health. 2025 Jan 3;5(1):e0002113. doi: 10.1371/journal.pgph.0002113 (PMC11698395; doi:10.1371/journal.pgph.0002113)
Supplement: Attachment — Submitted filename: Rebbutal to PLOS Global Health(2)- Final.pdf [file pgph.0002113.s001.pdf]

Dear Editorial team at PLOS Global Public Health,

We were pleased to have an opportunity to revise our manuscript now entitled “Copying mechanisms among students with mental health disorders at Busitema University In the revised manuscript, we have carefully considered the reviewers’ comments and suggestions. As instructed, we have attempted to explain changes made in reaction succinctly to all comments.

Our responses are shown as text. The reviewers’ comments were constructive overall, and we are appreciative of such constructive feedback on our original submission.
After addressing the issues raised, we feel the quality of the paper is much improved.

Sincerely
Enid Kawala Kagoya
Corresponding Author

1: Comments /Journal Requirements

Journal requirement 1. Please provide additional details regarding participant consent. In the ethics statement in the Methods and online submission information, please ensure that you have specified (1) whether consent was informed and (2) what type you obtained (for instance, written or verbal, and if verbal, how it was documented and witnessed). If your study included minors, state whether you obtained consent from parents or guardians. Please include this information if the ethics committee waived the need for consent.

Response: Thank you so much for this wonderful observation. I wish to inform you that consent was sought from all the participants. The consent obtained was "written" and we had no minors in this study. I am attaching a copy of the consent form as requested.

Journal Requirement 2: If you are reporting a retrospective study of medical records or archived samples, please ensure that you have discussed whether all data were fully anonymised before you accessed them and/or whether the IRB or ethics committee waived the requirement for informed consent. If patients provided informed written consent to have data from their medical records used in research, please include this information.

Response: There was no waiver for consent, participant consent was obtained and I have uploaded the consent form for you to look over.

3. Please amend your detailed Financial Disclosure statement. This is published with the article. It must therefore be completed in full sentences and contain the exact wording you wish to be published.

a. State the initials, alongside each funding source, of each author to receive each grant. b. State what role the funders took in the study. If the funders had no role in your study, please state: "The funders had no role in study design, data collection and analysis, decision to publish, or preparation of the manuscript."c. If any authors received a salary from any of your funders, please state which authors and which funders.

If you did not receive any funding for this study, please simply state: "The authors received no specific funding for this work."

Response: Thank you so much for this wonderful feedback, this part of the manuscript has been amended as requested.

4. Please provide separate figure files in PDF format only and remove any figures embedded in your manuscript file. Please also ensure all files are under our size limit of 10MB.

Response: Thank you so much for this productive feedback, this has been solved

Comments to the Author

1. Does this manuscript meet PLOS Global Public Health's publication criteria? Is the manuscript technically sound, and does the data support the conclusions? The manuscript must describe methodologically and ethically rigorous research with conclusions that are appropriately drawn based on the data presented.

Reviewer #1: Yes

Reviewer #2: Partly

Response: Thank you so much for this wonderful feedback. While drafting the manuscript, we tried to follow guidelines from PLOS. We carefully looked through them before and after writing the manuscript.

2. Has the statistical analysis been performed appropriately and rigorously?

Reviewer #1: Yes

Reviewer #2: N/A

Response: Thank you reviewers for this feedback, ideally since it was a purely qualitative study, the statistical analysis is not applicable.

3. Have the authors made all data underlying the findings in their manuscript fully available (please refer to the Data Availability Statement at the start of the manuscript PDF file)? The PLOS Data policy requires authors to make all data underlying the findings described in their manuscript fully available without restriction, with rare exceptions. The data should be provided as part of the manuscript or its supporting information, or deposited to a public repository. For example, in addition to summary statistics, the data points behind means, medians and variance measures should be available. If there are restrictions on publicly sharing data—e.g. participant privacy or use of data from a third party—those must be specified.

Reviewer #1: Yes

Reviewer #2: No

Response: Thank you so much for this feedback, this is indicated in the PLOS data policy. We tried to explain how sensitive the data was however, it would only be available on request.

4. Is the manuscript presented in an intelligible fashion and written in Standard English? PLOS Global Public Health does not copyedit accepted manuscripts, so the language in submitted articles must be clear, correct, and unambiguous. Any typographical or grammatical errors should be corrected at revision, so please note any specific errors here.

Reviewer #1: Yes

Reviewer #2: No

Response: Thank you Reviewers for this comment, the team has re-read the manuscript several times to identify any form of errors. The manuscript was also assessed for plagiarism.

5**. Review comments to the Author**

Please use the space provided to explain your answers to the questions above. You may also include additional comments for the author, including concerns about dual publication, research

ethics, or publication ethics. (Please upload your review as an attachment if it exceeds 20,000 characters)

Reviewer #1: This is a useful manuscript but I would request the authors to make some changes to improve the scientific rigor.
1. How did the authors decide the number of participants? Were they targeting data saturation? The authors need to clarify this.

Response: Thank you so much for this concern. This study follows another quantitative study where we first assessed students for mental health disorders and for those who screened positive, we interacted with them to understand the factors contributing to the disorders and their copying mechanisms. However, at 42, we had reached a point of saturation and we had to stop otherwise we had over 70 students who were diagnosed with mental health disorders.

2. If the analysis was deductive, did the authors decide a prior what the themes were? Could the authors clarify this?

Response: Thank you so much for your concern, I wish to inform you that priori coding was done systematically and later ATLAS software was used to reorganize the codes.

3. There are several limitations. As this is a qualitative study, it's not certain whether the results are generalizable. The findings are from one medical college and one engineering college. The findings need to be verified using quantitative methods. The authors have used MINI. Which version MINI? MINI does not pick up all psychiatric disorders. So the authors cannot generalize their findings to everyone with a psychiatric disorder.

Response: Thank you so much for this observation. I wish to inform you that qualitative findings were reported in a separate paper. This manuscript concentrated on the feedback from those who were diagnosed with mental health disorders according to the Mini just to explore their coping mechanisms.
4. Could the authors use the checklists for qualitative studies to improve the rigour?

Response: Thank you so much for this kind observation, I wish to inform you that the SROBE checklist was used in assessing rigour.

5. Could the authors provide clarifications for the use of key informants for this study?

Response: Thank you so much for this concern, we used key informant interviews because the respondents were already identified after the assessment.

6. Could the authors provide details of the semi-structured guide?

Response: Thank you so much for this concern, I have attached a copy of the interview guide for you to look over.

Reviewer #2: The study is a good initiative, however, requires major revision.
1. The title of the study should be relooked. The study states that they had recruited students who had positive diagnoses according to MINI. However, a major part of the results do not depict that the coping strategies used by the students are to mitigate these said 'Mental Disorders', but rather their psychological distress due to academic stress, financial stress or relationship issues, which essentially may not qualify to be classified as mental disorders. So the study fails to tell us in the discussion or in the conclusion how this coping mechanism is different for a person with a mental disorder when compared to a person without a mental disorder or how the MINI assessment was significant for the study.

Response: Thank you so much for this concern. The team took off time to revise the whole paper and we have made the necessary changes. Just to inform you that the study recruited students who screened positive for mental health disorders. Those who did not have any were not part of the qualitative interviews.

2. The method used is sound and 42 interviews is absolutely a great sample. However, a major revision is needed in the themes as the themes and title don't match exactly. For example:

2. a. In the theme, opportunity, confrontation and abortion, the verbatim depicted the challenges faced by the students rather than their coping mechanisms.

Response: Thank you so much for this concern. Under that theme, we meant that most of the students opted for confrontation and abortion as a way to cope with mental health challenges. Like some of the students who ended up conceiving during their relationships, abortion was an alternative that set them, free and some of them opted for confrontation with their lecturers and fellow stents as a way of peeling off the anger and anxiety.

2. b. In the theme Withdrawal, the theme withdrawal from relationships, the reviewer fails to understand whether the relationship had anything to do with their mental disorder or how they used it as a coping mechanism for their mental disorder.

Response: Thank you so much for this observation. By withdrawal, students opted to withdraw from the relationships because they were contributing to their state of mental well-being.

Response: Thank you so much for this concern. By withdrawal, Students had to withdraw from relationships because they were so sophisticated, and complicated contributing to their mental well-being.

2. c. The sub-theme, use of witchcraft, again was to alleviate their financial struggles or difficulties with relationships or to be successful in business, but not of mental health issues. None of the verbatim supported the idea that the respondents used witchcraft to reduce their concerns related to mental disorders/mental health concerns, unlike the usual pathway to care of using witchcraft to reduce the distress due to the symptoms of mental disorders.

Response: Thank you so much for this concern however witchcraft was a coping mechanism, especially to those who had financial and relationship challenges. As a way of securing their finances which they were struggling to get from small businesses, witchcraft was an option as well as for securing their relationship with their girlfriends. Most of them who opted to use it informed us that they had competitions in their businesses which were earning them tuition, so for them to calm down the competition so that they also win the hearts of customers, witchcraft was a solution. Then for boys who didn’t want their gal friends to be taken, witchcraft was an alternative as it

would make them stick to them and not any other person and in the long run solve their mental health challenges.

2.d. The sub-theme, Using alcohol, the second verbatim says about social drinking, but not a coping mechanism.

Response: Thank you so much for your response, social drinking was a coping strategy for them especially amidst pressure for exams and unfavourable tests and exams, most of them informed us that when they were depressed, social during was a way to cope would keep in there and forget their problems or mental health challenges.

3. The study should be reworked grammatically including punctuation, spellings etc. For example, in the short title it is mentioned as Copying instead of Coping, throughout the text, Campus is misspelt as Compass, Peels instead of Pills.

Response: Thank you so much for this concern, a thorough grammatical check has been conducted and all the grammar errors have been solved.

4. While considering the ethical part of the study, the students are positively diagnosed with psychiatric disorders. Were they already on treatment? If not, when the research team, assessed them for psychiatric disorders, did they refer them to the appropriate treatment facility? What was the SOP when you interviewed the respondents with suicidal ideations?

Response: Thank you so much for this observation however, none of the students was on treatment at the time of the study. This was a random study done at both compasses, we screened all the students and only those who had mental health disorders were interviewed on their coping mechanisms otherwise no one was on treatment at the time we conducted the study.

For those with suicidal behavior, they were interviewed by the university counsellor and psychiatrists however they expressed willingness to participate in the study.`

6. PLOS authors have the option to publish the peer-reviewed history of their article (what does this mean?). If published, this will include your full peer review and any attached files.

Do you want your identity to be public for this peer review? If you choose “no”, your identity will remain anonymous but your review may still be made public.

Thank you so much for your concern, I have no objection to that. Feel free to do as required.

For information about this choice, including consent withdrawal, please see our Privacy Policy.

Reviewer #1: **Yes:** priya sreedaran

Reviewer #2: No

Response

Thank you so much for all the comments, I agree and permit full consent to the journal to use my work.

Thank you so much
