# Supplementary material for: Coping mechanisms for students with mental disorders: An exploratory qualitative study at Busitema University’s Mbale and Busia Campuses
Source: PLOS Glob Public Health. 2025 Jan 3;5(1):e0002113. doi: 10.1371/journal.pgph.0002113 (PMC11698395; doi:10.1371/journal.pgph.0002113)
Supplement: Attachment — Submitted filename: Rebbutal to PLOS Global Health 15 Manuscript.docx [file pgph.0002113.s003.docx]

13^th^/7/2024

[enidkawala@gmail.com](mailto:enidkawala@gmail.com)

Busitema University

Faculty of Health Sciences

The Editorial Team

PlOS Global Public Health

Dear Editorial team at PLOS Global Public Health,

We were pleased to have an opportunity to revise our manuscript now entitled
“Copying mechanisms among students with mental health disorders at Busitema University In the revised manuscript, we have carefully considered the reviewers’ comments and suggestions. As
instructed, we have attempted to explain changes made in reaction succinctly
to all comments.

Our responses are shown as text. The reviewers’ comments were constructive overall, and we are
appreciative of such constructive feedback on our original submission.

After addressing the issues raised, we feel the quality of the paper is much improved.

Sincerely
Enid Kawala Kagoya

Corresponding Author

**Comment**

1. Please provide additional details regarding participant consent. In the ethics statement in the Methods and online submission information, please ensure that you have specified (1) whether consent was informed and (2) what type you obtained (for instance, written or verbal, and if verbal, how it was documented and witnessed).

**Response: The informed consent was written and each participant signed the informed consent document in the presence of a third person who was not part of the study team as a witness. This has been provided in the manuscript.**

2. Please ensure you have also stated whether you obtained consent from parents or guardians of the minors included in the study or whether the research ethics committee or IRB specifically waived the need for their consent.

**Response: This study interviewed participants who were 18 years and above, the legal age limit for consent in the study country, Uganda. No minors were interviewed as such there was no new need for a third-party consent or waiver for informed consent from the ethics committee. We have provided this statement “There was no need for a third party consent” in the manuscript to cater for this.**

Thank you so much

Enid Kawala Kagoya

Corresponding Author
