# Supplementary material for: Coping mechanisms for students with mental disorders: An exploratory qualitative study at Busitema University’s Mbale and Busia Campuses
Source: PLOS Glob Public Health. 2025 Jan 3;5(1):e0002113. doi: 10.1371/journal.pgph.0002113 (PMC11698395; doi:10.1371/journal.pgph.0002113)
Supplement: Attachment — Submitted filename: POINT BY POINT RESPONSE - PLOS Global Health 1.docx [file pgph.0002113.s004.docx]

**POINT-BY-POINT RESPONSE**

| **No** | **PLOS Comment** | **Response** | **Liner number (s) in tracked changes** | **Page number (s) in tracked changes** | **Liner number (s) in clean copy** | **Page number (s) in clean copy** |
| --- | --- | --- | --- | --- | --- | --- |
| 1 | Please provide additional details regarding participant consent. In the ethics statement in the Methods and online submission information, please ensure that you have specified. | Thank you so much for this response, this has been done This has been amended as indicated | 109 | 6 | 109 | 6 |
| 2 | Whether consent was informed and what type you obtained (for instance, written or verbal, and if verbal, how it was documented and witnessed) | Thank you so much for this response, this has been done This has been amended as indicated | 122-123 | 5 | 123 | 5 |
| 3 | If your study included minors, state whether you obtained consent from parents or guardians. If the need for consent was waived by the ethics committee, please include this information. | Ethical approval was obtained from the Research Ethics Committee (REC) of Mbale Regional Referral Hospital (Approval No. MRRH-2023-300). Written informed consent was obtained from all participants, with the consent form approved by the REC | 139-146 | 6 | 140-144 | 6 |
| 4 | If you are reporting a retrospective study of medical records or archived samples, please ensure that you have discussed whether all data were fully anonymised before you accessed them and/or whether the IRB or ethics committee waived the requirement for informed consent. | Not applicable | Not Applicable | 6 |  |  |
| 5 | If patients provided informed written consent to have data from their medical records used in research, please include this information | Thank you so much for this response, this has been done This has been amended. | 109 | 6 | 110 | 6 |
| 5 | There is a need for rigour in this submission before it is suitable for publication. In qualitative studies, the reliability of methods is an important aspect. The authors have not conveyed their qualitative analysis | Thank you so much for this response, this has been done This has been amended. | 129-137 | 6 | 128-137 | 6 |
| 6 | They have mentioned thematic analysis and deductive approach. However, if the authors were to read the approaches to thematic analysis, they would realize the current description is not enough. | Thank you so much for this response, this has been done This has been amended. | 114-129 | 6 | 114-129 | 6 |
| 7 | They need to specify who did the coding, did they depended entirely on NVivo, who did the entry into NVivo, how they derived codes, and how they extrapolated themes from these. | Thank you so much for this response, this has been done This has been amended. | 116-127 | 6 | 116-129 | 6 |
|  | This discussion on coding is missing. Did the authors use a more reflexive thematic approach or a codebook type of approach? | Thank you so much for this response, this has been done This has been amended. | 116-124 | 6 | 120-124 | 6 |
