# Supplementary material for: Coping mechanisms for students with mental disorders: An exploratory qualitative study at Busitema University’s Mbale and Busia Campuses
Source: PLOS Glob Public Health. 2025 Jan 3;5(1):e0002113. doi: 10.1371/journal.pgph.0002113 (PMC11698395; doi:10.1371/journal.pgph.0002113)
Supplement: Attachment — Submitted filename: POINT BY POINT RESPONSE - PLOS Global Health Vr 16.11.docx [file pgph.0002113.s005.docx]

**POINT-BY-POINT RESPONSE**

**Submitted to PLos Global Health**

**22/10/2024**

| **No** | **PLOS Comment** | **Response** | **Liner number (s) in tracked changes** | **Page number (s) in tracked changes** | **Liner number (s) in clean copy** | **Page number (s) in clean copy** |
| --- | --- | --- | --- | --- | --- | --- |
| 1 | Please provide additional details regarding participant consent. In the ethics statement in the Methods and online submission information, please ensure that you have specified (1) whether consent was informed and (2) what type you obtained (for instance, written or verbal, and if verbal, how it was documented and witnessed). If your study included minors, state whether you obtained consent from parents or guardians. If the need for consent was waived by the ethics committee, please include this information. | Informed consent was sought as revised. | 108 | 4 | 108 | 4 |
| 2 | Whether consent was informed and what type you obtained (for instance, written or verbal, and if verbal, how it was documented and witnessed) | Thank you so much for this response, this has been done This has been amended as indicated | 108 | 4 | 108 | 4 |
| 3 | If your study included minors, state whether you obtained consent from parents or guardians. If the need for consent was waived by the ethics committee, please include this information. | Ethical approval was obtained from the Research Ethics Committee (REC) of Mbale Regional Referral Hospital (Approval No. MRRH-2023-300). Written informed consent was obtained from all participants, with the consent form approved by the REC  The study did not include minors and no waiver was sought from the IRB | 137-140 | 5 | 137-140 | 5 |
| 4 | If you are reporting a retrospective study of medical records or archived samples, please ensure that you have discussed whether all data were fully anonymised before you accessed them and/or whether the IRB or ethics committee waived the requirement for informed consent. | Not applicable because we are not reporting data from medical records | N/A | N/A | N/A | N/A |
| 5 | Version of MINI used | We used MINI version 7.0.2 | 108 | 4 | 108 | 4 |
| 6 | Please review your reference list to ensure that it is complete and correct. If you have cited papers that have been retracted, please include the rationale for doing so in the manuscript text, or remove these references and replace them with relevant current references. Any changes to the reference list should be mentioned in the rebuttal letter that accompanies your revised manuscript. If you need to cite a retracted article, indicate the article’s retracted status in the References list and also include a citation and full reference for the retraction notice. | Thank you so much for this observation, the reference list has been revised  These have been revised | 404-442  45-78  481-583 | 2-3  16-17  19-20 | 404-442  45-78  481-583 | 2-3  16-17  19-20 |
