# Supplementary material for: Coping mechanisms for students with mental disorders: An exploratory qualitative study at Busitema University’s Mbale and Busia Campuses
Source: PLOS Glob Public Health. 2025 Jan 3;5(1):e0002113. doi: 10.1371/journal.pgph.0002113 (PMC11698395; doi:10.1371/journal.pgph.0002113)
Supplement: Attachment — Submitted filename: POINT BY POINT RESPONSE - PLOS Global Health Vr 16.12.docx [file pgph.0002113.s006.docx]

**POINT-BY-POINT RESPONSE**

| **No** | **PLOS Comment** | **Response** | **Liner number (s) in tracked changes** | **Page number (s) in tracked changes** | **Liner number (s) in clean copy** | **Page number (s) in clean copy** |
| --- | --- | --- | --- | --- | --- | --- |
| 1 | Which version of MINI did the authors use? | We used MINI version 7.0.2 | 108 | 4 | 108 | 4 |
| 2 | How did the authors develop the semi-structured interviews? | The Semi structured interview guide was based on the research questions and also existing literature  However the guide was formulated by a team of experts, we developed a semi structured interviewer guide which was pretested on a given number of students making it suitable for the data collection.  The technical team included the psychiatrists, a psychologist, an academicians, a behavioural scientists and the university counsellor. | N/A | N/A | N/A | N/A |
| 3 | What were the initial domains anticipated as part of the deductive analysis? Did the generated themes meet these domains? | Thank you for this wonderful observation. I wish to inform you that that was oversight  We actually used inductive approach guided by specific objectives as corrected on line 112 on page 5 | 112 | 5 | 112 | 5 |
