# Supplementary material for: Coping mechanisms for students with mental disorders: An exploratory qualitative study at Busitema University’s Mbale and Busia Campuses
Source: PLOS Glob Public Health. 2025 Jan 3;5(1):e0002113. doi: 10.1371/journal.pgph.0002113 (PMC11698395; doi:10.1371/journal.pgph.0002113)
Supplement: Attachment — Submitted filename: Rebbutal letter- PLOS Global Health Vr 16.13 r.docx [file pgph.0002113.s007.docx]

Busitema University, faculty of health sciences

P.O Box, 1460, Mbale

16^th^/November/2024

The Editorial Committee

PLOS Global Health

Dear Reviewers

**RE: Rebuttal for manuscript Coping Mechanisms for Students with Mental Disorders: An Exploratory Qualitative Study at Busitema University’s Mbale and Busia Campuses**

We would like to thank the reviewers and editors for your thoughtful and constructive comments on our manuscript. Your feedback has been invaluable in improving the quality of the manuscript. In response to the your suggestions, we have revised several sections of the manuscript, including clarifying the type of consent obtained, the reference lists and the use of retrospectively using medical records as summarized in the table below.

| **No** | **PLOS Comment** | **Response** | **Liner number (s) in tracked changes** | **Page number (s) in tracked changes** | **Liner number (s) in clean copy** | **Page number (s) in clean copy** |
| --- | --- | --- | --- | --- | --- | --- |
| 1 | Please provide additional details regarding participant consent. In the ethics statement in the Methods and online submission information, please ensure that you have specified (1) whether consent was informed and (2) what type you obtained (for instance, written or verbal, and if verbal, how it was documented and witnessed). If your study included minors, state whether you obtained consent from parents or guardians. If the need for consent was waived by the ethics committee, please include this information. | Thank you so much for this kind observation.  This has been included because we obtained a “written informed consent” from all participants as guided by the Research Ethics Committee and guidelines for research involving individual subjects.  I wish to inform you that the study included students who were above 18 years with no disability therefore there were no minors in this study.  No waiver of consent was obtained because all the participants (students) consented to participate in the study. | 140-142 | 5-6 | 140-142 | 5-6 |
| 2 | If you are reporting a retrospective study of medical records or archived samples, please ensure that you have discussed whether all data were fully anonymized before you accessed them and/or whether the IRB or ethics committee waived the requirement for informed consent. If patients provided informed written consent to have data from their medical records used in research, please include this information. | Thank you so much for this feedback  I wish to inform you that data was collected prospectively using key informant interviews and no medical records were used therefore the study does not report any retrospective data from medical records or achieved samples. | N/A | N/A | N/A | N/A |
| 3 | Please review your reference list to ensure that it is complete and correct. If you have cited papers that have been retracted, please include the rationale for doing so in the manuscript text, or remove these references and replace them with relevant current references. Any changes to the reference list should be mentioned in the rebuttal letter that accompanies your revised manuscript. If you need to cite a retracted article, indicate the article’s retracted status in the References list and also include a citation and full reference for the retraction notice. | Thank you so much for the kind comments.  The reference list has been revised and all retracted papers have been replaced as indicated in reference 14, 16.17,18 and 19. | 18,52  423,439,440  481-484,489-490, 519-535 | 2, 16-17  19-20 | 18,52  423,439,440  481-484,489-490, 519-535 | 2, 16-17  19-20 |
| 4 | While revising your submission, please upload your figure files to the Preflight Analysis and Conversion Engine (PACE) digital diagnostic tool, <https://pacev2.apexcovantage.com/>. PACE helps ensure that figures meet PLOS requirements. To use PACE, you must first register as a user. Registration is free. Then, login and navigate to the UPLOAD tab, where you will find detailed instructions on how to use the tool. If you encounter any issues or have any questions when using PACE, please email PLOS at [figures@plos.org](mailto:figures@plos.org). Please note that Supporting Information files do not need this step. | Thank you so much for this comment  I wish to inform you that this paper has no figures, its only tables. | N/A | N/A | N/A | N/A |

Once again, we thank the reviewers for their helpful comments. We believe that the revisions have significantly improved the manuscript, and we look forward to your feedback on the revised version.

Yours Sincerely

Enid Kawala Kagoya

Corresponding Author
