# Supplementary material for: Coping mechanisms for students with mental disorders: An exploratory qualitative study at Busitema University’s Mbale and Busia Campuses
Source: PLOS Glob Public Health. 2025 Jan 3;5(1):e0002113. doi: 10.1371/journal.pgph.0002113 (PMC11698395; doi:10.1371/journal.pgph.0002113)
Supplement: Attachment — Submitted filename: POINT BY POINT RESPONSE - PLOS Global Health Vr 16.14.docx [file pgph.0002113.s008.docx]

**POINT-BY-POINT RESPONSE**

**Submitted to PLOS Global Health**

**23/11/2024**

| **No** | **PLOS Comment** | **Response** | **Liner number (s) in tracked changes** | **Page number (s) in tracked changes** | **Liner number (s) in clean copy** | **Page number (s) in clean copy** |
| --- | --- | --- | --- | --- | --- | --- |
| 1 | Version of MINI used | Thank you so much for this observation,  I wish to inform you that we used MINI version 7.0.2 as indicated in the manuscript | 105 | 4 | 105 | 4 |
| 2 | How did the authors develop the semi structured interviews | Thank you so much for this observation  To develop semi-structured interviews, we typically followed a systematic approach that included several key steps.  We first established the primary goals of the research, identifying the key themes or topics that we aimed to explore through the interview. These objectives guided the creation of questions that align with the purpose of the study.  We as well conducted some literature review to understand what other studies had explored regarding the same or related topics. This helped us identify gaps in the literature and areas that need further investigation.  Based on the research objectives and literature, we determined the core themes to explore. These themes were broad (e.g., personal experiences, attitudes, behaviors, or perceptions towards preeclampsia)  We went ahead to create our interview guide**.** We drafted a set of open-ended questions related to the identified themes.  We also included prompts or follow-up questions to encourage further elaboration, helping us to dive deeper into responses.  The questions were designed to be flexible, allowing us to follow up on interesting points raised by the interviewee during the conversation.  We conducted a pilot test with a small sample to refine the interview guide. This allowed us to check for clarity, relevance, and appropriateness of the questions. Adjustments were made based on feedback and observations during these preliminary interview.  We also ensured that the interview guide respects ethical principles, such as confidentiality, informed consent, and the right to withdraw from the study. | N/A | N/A | N/A | N/A |
| 3 | What were the initial domains anticipated as part of the deductive analysis? Did the generated theme meet these domains | Thank you so much for this observation  I wish to inform you that this was an oversight. We used an inductive approach as corrected in the manuscript.  We began with broad, open-ended categories or domains that we anticipated might emerge from the data, without imposing predefined structures.  These initial domains were based on the research questions and the overarching themes that the study explored.  The themes kept emerging naturally from the data through participant responses, rather than being imposed upfront. | 112 | 5 | 112 | 5 |
