# Supplementary material for: Coping mechanisms for students with mental disorders: An exploratory qualitative study at Busitema University’s Mbale and Busia Campuses
Source: PLOS Glob Public Health. 2025 Jan 3;5(1):e0002113. doi: 10.1371/journal.pgph.0002113 (PMC11698395; doi:10.1371/journal.pgph.0002113)
Supplement: Attachment — Submitted filename: POINT BY POINT RESPONSE - PLOS Global Health Vr 16.15.docx [file pgph.0002113.s009.docx]

**POINT-BY-POINT RESPONSE**

**Submitted to PLOS Global Health**

**27/11/2024**

| **No** | **PLOS Comment** | **Response** | **Liner number (s) in tracked changes** | **Page number (s) in tracked changes** | **Liner number (s) in clean copy** | **Page number (s) in clean copy** |
| --- | --- | --- | --- | --- | --- | --- |
| 2 | How did the authors develop the semi structured interviews | Thank you so much for this observation, this has been summarises as follows and included in the manuscript as well.    To develop semi-structured interviews, we began by defining the research goals and identifying key themes to be explored.  A literature review was done to help us identify gaps and refine the areas of focus. We then created an interview guide with open-ended questions and follow-up prompts based on these themes, ensuring flexibility for deeper exploration.  A pilot test with a small sample allowed us to refine the guide for clarity and relevance.  Throughout, we maintained ethical standards, including confidentiality and informed consent. | 109-115 | 4-5 | 109-115 | 4-5 |
